# Supplementary material for: Global assessment of existing HIV and key population stigma indicators: A data mapping exercise to inform country-level stigma measurement
Source: PLoS Med. 2022 Feb 22;19(2):e1003914. doi: 10.1371/journal.pmed.1003914 (PMC8903269; doi:10.1371/journal.pmed.1003914)
Supplement: S1 Protocol — (DOCX) [file pmed.1003914.s001.docx]

**Protocol: Indices on stigma and discrimination related to HIV and key populations – Phase 1**

Given the demonstrated impact of stigma and discrimination on HIV transmission, prevention, and treatment outcomes, there is a critical need for measures that can characterize the level and nature of stigma and discrimination in a given country and assess progress towards eliminating these barriers to achieving the end of the HIV/AIDS epidemic by 2030. In order to meet the goal laid out by UNAIDS of developing five index measures of stigma and discrimination- one each related to HIV, sex work, injection drug use, being a man who has sex with men and transgender- we propose a four-phase approach. The phases we propose are as follows:

1. Indicator finalization and data mapping
2. Composite index development
3. Piloting and validation
4. Index revision and dissemination

The purpose of this protocol is to describe a methodology and technical specifications for the development of these five potential summary indices, i.e., how to combine the proposed indicators, domains, and subdomains into a valid and useful measure of stigma and discrimination inclusive of the policy level within a country, and to present preliminary results on weighting and aggregation of indicators. This section of the protocol includes a description of activities related to phase 1 of this study: indicator finalization and data mapping.

**1. Indicator finalization and data mapping**

In collaboration with UNAIDS, the goal of Phase 1 is to determine the number of countries which have data to calculate each indicator over the period of 2000-2020. We will also gather up to date information to report on the data source and availability for each potential indicator proposed by UNAIDS.

***1A. Preparation for data mapping***

*1A.1. Initial drafting of potential domains, subdomain, and indicators*

The UNAIDS Science Panel held a meeting in 2017 that resulted in a recommendation to create measures of the legal and policy environment as it relates to the reduction of stigma and discrimination in order to facilitate progress towards meeting the 2030 Agenda for Sustainable Development goal (SDG 3.3) of achieving the end of the HIV pandemic. The UNAIDS Monitoring Technical Advisory Group (MTAG) then sought to build upon these recommendations in 2018 through the creation of a task team of civil society and technical experts on HIV and key population stigma from around the globe to being to operationalize country-level summary measures of stigma and discrimination related to HIV and four key populations: men who have sex with men, sex workers, injection drug users, and trans people. UNAIDS created a draft set of domains, subdomains and indicators of HIV and key population stigma for this purpose and received feedback on these domains from stakeholders through a 3-week e-consultation in August-September 2019.

- Outcome: Drafted potential domains, subdomains, and indicators using existing measures.

*1A.2. Review of proposed domains, subdomain, and indicators*

- The UNAIDS Monitoring Technical Advisory Group (MTAG) built upon recommendations in 2018 through the creation of a task team of civil society and technical experts on HIV and key population stigma from around the globe that began to operationalize country-level summary measures of stigma and discrimination related to HIV and four key populations: men who have sex with men, sex workers, injection drug users, and trans people.
- A task team of experts across multilateral agencies, academia, and community will review and will provide feedback to the initial selection of domains, subdomains, and indicators drafted by UNAIDS.
- Outcome: Updated draft set of domains, subdomains and indicators of HIV and key population stigma with feedback from experts in the field

*1A.3. E-consultation*

- Task team will lead a 3-week e-consultation to obtain feedback on the domains, subdomains, and indicators drafted by UNAIDS.
- At the stage of drafting, no limitation will be placed on the inclusion of an indicator, sub-domain or domain based on whether data was available through regular, repeat data collection and/or compiled in a repository or global database. This is the rationale behind the first phase of this consultancy process- to first conduct a data mapping exercise to fully assess the level of available data for the creation of summary measures.
- The consultation information will be shared through UNAIDS mailing lists, social media, and partners.
- Interested participants were invited to register on the consultation page. No limitation will be placed on participation.
- Outcome: Feedback from a larger group of stakeholders on the proposed domains, subdomains, and indicators drafted by UNAIDS

***1B. Mapping available data for country-level summary measures of HIV and key population stigma***

Regular meetings will be held by the stigma index creation/analysis team to plan and implement data mapping, discuss challenges, and create decision points for identified data.

*1B.1 Institutional Review Board for Research with Human Subjects.*

- The data mapping exercise will be reviewed by the JHU Institutional Review Board (IRB). JHU will submit a description of the planned data mapping exercise for initial review for human subjects research. The IRB may determine that this work is non-human subjects research and does not need to have oversight from the IRB. Conversely, the IRB may require this work be managed as secondary data analysis with continued oversight. In case of the later, JHU has an existing protocol approved by the IRB for the secondary analysis of existing datasets to establish metrics of stigma and discrimination related to HIV and key populations. JHU will submit these planned analyses as an amendment to this protocol which would allow us to access any de-identified non-public use data required for this consultancy and to publish the results of our analyses of those data efficiently.

*1B.2. Indicator identification and extraction*

- The data mapping exercise is an exploratory process. Specific criteria for inclusion and exclusion will guide the process; however, final cut points for these criteria will be determined based on the data obtained during the mapping process.
- The data mapping team will acquire relevant datasets in collaboration with UNAIDS. We would proceed to request all necessary individual and country level variables from the datasets identified as relevant in the data mapping exercise. Based on preliminary review of the proposed indicators, we would expect this to include data from the following sources: UNAIDS Commitments and Policies Instrument (NCPI) database; Multiple Indicator Cluster Surveys (MICS); Demographic and Health Surveys (DHS); People Living with HIV Stigma Index 1.0 surveys; HIV Key Population Data Repository; a selection of Integrated Biological and Behavioral Surveys (IBBS) and, any studies where the People Living with HIV Stigma Index 1.0 was administered.
- During this process, the team will also note any potential indicators for which there is data available that may be relevant to the existing domains and subdomains for stigma related to the indices under development. These will be proposed as potential additions.
- The team will set up a combined database that includes data for all indicators from the sources identified in the section 1A and conduct exploratory data analyses. Exploratory data analysis will include assessment of missing data and further data cleaning (e.g., reverse coding) prior to indicator creation. We would then calculate summary statistics (i.e., means, frequencies, variances, ranges) for all indicators to determine if any data transformations are necessary, any outlier values need to be managed, and whether sufficient variation in values exists for use in a composite index. If the majority of indicators are binary, we will determine the best cut-offs to use to transform continuous variables into binary indicators.
- We will then generate a correlation matrix of all indicator variables to assess redundancy. While we expect correlation between the indicators, a very high degree of correlation between two indicators would suggest redundancy that should be eliminated to create the most parsimonious and informative measure. At this stage, we will also develop an approach for dealing with missing data depending on the extent of missing data and the assumptions that can be made about the nature of missingness for the indicators that are included. Unless key assumptions are not met or the extent of missing data is small, we anticipate using a multiple imputation approach to address bias without artificially deflating variance.

*1B.3. Indicator selection based on a priori criteria*

- Indicators for which no existing public or multiple country data source could be identified will be excluded.
- Indicators for which data were available from less than a certain number of countries will be excluded. This cut point will be determined based on the data available.
- Indicators with <10% endorsement will be considered to have low variation and considered for exclusion or potential combination.
- Indicators presenting fundamental concerns in the response options, data collection methods (e.g., collected and weighted separately for subgroups such that the data cannot be combined without access to individual level data), or representativeness will be considered for exclusion.
- Indictaors with high levels of correlation will be considered for exclusion given considerations of redundancy.

*1B.4. Indicator assessment based on a priori criteria*

- Indicators will be categorized based on the following criteria:
  1. Proportion of countries for which data were available.
  2. Level of missingness of data within countries.
  3. Potential for calculation of the indicator over time.

*1B.5. Review*

- Our workplan and timeline will be revised as necessary based on results of activities 1B, after consulting with UNAIDS on a workshopping call. At this time, further revisions to the analysis plan can be made based on additional expert feedback UNAIDS has received, and the results of data mapping and exploration exercises prior to proceeding to phase 2.
