# Supplementary material for: Global assessment of existing HIV and key population stigma indicators: A data mapping exercise to inform country-level stigma measurement
Source: PLoS Med. 2022 Feb 22;19(2):e1003914. doi: 10.1371/journal.pmed.1003914 (PMC8903269; doi:10.1371/journal.pmed.1003914)
Supplement: S1 Table — (DOCX) [file pmed.1003914.s002.docx]

**S1 Table. Characteristics of participants in E-consultation**

| **Geographic region of work** | **%** | **N** |
| --- | --- | --- |
| Asia and the Pacific | 22.43% | 24 |
| Caribbean | 4.67% | 5 |
| Eastern Europe and Central Asia | 5.61% | 6 |
| Eastern and Southern Africa | 26.17% | 28 |
| Latin America | 3.74% | 4 |
| Middle East and North Africa | 8.41% | 9 |
| Western and Central Africa | 14.95% | 16 |
| Western and Central Europe and North America | 40.19% | 43 |
| **Sector of work** |  |  |
| Affected community | 21.50% | 23 |
| Community-based organization | 27.10% | 29 |
| Faith-based organization | 1.87% | 2 |
| Civil society organization | 19.63% | 21 |
| Non-governmental organization | 39.25% | 42 |
| Private sector | 7.48% | 8 |
| Foundation | 2.80% | 3 |
| Academia | 16.82% | 18 |
| Parliament | 0.93% | 1 |
| Government ministry | 5.61% | 6 |
| Professional organization | 8.41% | 9 |
| Other (please specify) | 10.28% | 11 |
