# Supplementary material for: Global assessment of existing HIV and key population stigma indicators: A data mapping exercise to inform country-level stigma measurement
Source: PLoS Med. 2022 Feb 22;19(2):e1003914. doi: 10.1371/journal.pmed.1003914 (PMC8903269; doi:10.1371/journal.pmed.1003914)
Supplement: S2 Table — (DOCX) [file pmed.1003914.s003.docx]

**S2 Table. Proposed indicators for HIV stigma**

| **Domain** | **Sub-Domain** | **Indicator** | **Included/**  **Excluded** | **Rationale for exclusion** |
| --- | --- | --- | --- | --- |
| Norms and attitudes | Discriminatory attitudes towards people living with HIV | Percentage of women and men 15-49 years old who report discriminatory attitudes | Included |  |
|  |  | Percentage of health facility staff that hold stigmatizing views | Excluded | Limited number of countries with data. |
|  | Perceived HIV-related stigma | Percentage of people who report negative individual- and population-level manifestations of HIV-related stigma | Excluded | Limited number of countries with data. No planned data collection |
|  | HIV-related stereotypes | Percentage of people who report fear of HIV infection through non-invasive contact with a person living with HIV | Excluded | Limited number of countries with data. No planned data collection |
|  |  | Percent of health facility staff who worry about getting HIV when providing care or services to patients living with HIV | Excluded | Limited number of countries with data. No planned data collection |
|  | Acceptability of partner violence | Percentage of all women and men who agree that a husband is justified in hitting or beating his wife for specific reasons | Included |  |
| Structural stigma | Criminalization of HIV | Existence of laws criminalizing vertical transmission | Excluded | Low variation |
|  |  | Existence of laws criminalizing the transmission of, non-disclosure of, or exposure to HIV transmission | Included |  |
|  |  | Number of prosecutions for HIV transmission | Included |  |
|  | Restriction on entry, stay, or residence | Existence of laws restricting the entry, stay and residence of people living with HIV | Included |  |
|  | Mandatory testing | Existence of laws, regulations or policies specifying HIV testing is mandatory before marriage, to obtain a work or residence permit and/or for certain groups | Included |  |
|  | Consent to access sexual and reproductive health and HIV services | Existence of laws requiring parental/guardian consent for adolescents to access HIV testing and receive the results | Included |  |
|  |  | Existence of laws requiring parental/guardian consent for adolescents to access HIV treatment | Included |  |
|  |  | Existence of laws requiring parental/guardian consent for adolescents to access contraceptives, including condoms | Included |  |
|  |  | Existence of laws requiring spousal consent for married women to access any sexual or reproductive health service | Included |  |
|  |  | Existence of laws requiring spousal consent for married women to access HIV testing | Excluded | Low variation |
|  | Non-discrimination | Existence of laws or policies requiring healthcare settings to provide timely and quality healthcare regardless of any grounds | Included |  |
|  |  | Existence of laws protecting against discrimination on the basis of HIV status | Included |  |
|  |  | Percent of health facility staff who report that their facility has written guidelines to protect patients living with HIV from discrimination | Excluded | Limited number of countries with data. No planned data collection |
|  |  | Training programmes for specific groups on human rights and non-discrimination legal frameworks as applicable to HIV | Excluded | No data available |
|  |  | Existence of training and/or capacity-building programmes for people living with HIV and key on their rights (in the context of HIV) | Excluded | No data available |
|  |  | Percentage of educational institutions that have rules and guidelines for staff and students related to physical safety, stigma and discrimination and sexual harassment and abuse that have been communicated to relevant stakeholders | Excluded | No data available |
|  |  | Percentage of workplaces that have non-discriminatory policies that address HIV status, gender equality, and sexual orientation | Excluded | No data available |
|  | Access to Justice | Existence of mechanisms to access affordable legal services/Existence of government mechanisms to record and address individual complaints cases of HIV-related discrimination (based on perceived HIV status and/or belonging to any key population) | Excluded | Low variation |
|  |  |  | Included |  |
|  |  | Existence of accountability mechanisms in relation to discrimination and violations of human rights in healthcare settings | Excluded | Systematic concerns over data quality or accuracy |
| Violence (physical, sexual, emotional/ psychological, economic) | Controlling partner behaviors | Percentage of ever-married women whose husbands/partners demonstrated types of controlling behaviors | Included |  |
|  | Recent experience of violence | Percentage of women age 15-49 who have experienced physical and/or sexual violence by an intimate partner | Included |  |
|  |  | Percentage of women age 15-49 who have experienced physical and/or sexual violence by someone other than an intimate partner in the past 12 months | Excluded | Systematic concerns over data quality or accuracy |
|  | National policy environment | Existence of a national plan or strategy to address gender-based violence and violence against women that includes HIV | Included |  |
|  |  | Existence of specific legal provisions prohibiting violence against people based on their HIV status or belonging to a key population | Included |  |
|  | Experience of violence in healthcare settings | Percentage of people living with HIV who were forced, pressured or paid/incentivized to get sterilized and/or advised to terminate a pregnancy | Included |  |
| Anticipated and experienced stigma | Social exclusion | Percentage of people living with HIV who experienced social exclusion in the last 12 months due to their HIV status | Included |  |
|  | Discrimination experienced in healthcare settings | Percentage of people living with HIV who report experiences of HIV-related discrimination in healthcare settings | Included |  |
|  | Discrimination anticipated in healthcare settings | Percentage of people living with HIV who avoided seeking healthcare in the past 12 months due to fear of stigma and discrimination | Included |  |
|  | Discrimination experienced in employment | Percentage of people living with HIV who have lost a source of income or job because of their HIV status in the past 12 months | Included |  |
|  |  | Percentage of people living with HIV who have been refused employment or a work opportunity because of their HIV status in the past 12 months | Included |  |
|  | Discrimination experienced related to housing | Percentage of people living with HIV who have been forced to move or unable to rent a place to live because of their HIV status in the last 12 months | Excluded | No data available |
|  | Discrimination experienced in education settings | Cases of bullying in school (indirectly or directly link | Excluded | No data available |
|  |  | Anticipated stigma in education settings, i.e. fear | Excluded | No data available |
|  | Discrimination experienced in private services (e.g., insurance, banking loans) | Unclear | Excluded | No data available |
| Internalized stigma | Social isolation | Percentage of people living with HIV that report self-isolating from others | Included |  |
|  | Negative self-beliefs or feelings | Percentage of people living with HIV that report shame or guilt | Included |  |
|  | Self-harm, suicide attempts, or self-diminishing actions | Percentage of people living with HIV that report feeling suicidal due to their HIV status | Excluded | No data available |
|  | Self-diminishing feelings | TBD | Excluded | No data available |
