# Supplementary material for: Global assessment of existing HIV and key population stigma indicators: A data mapping exercise to inform country-level stigma measurement
Source: PLoS Med. 2022 Feb 22;19(2):e1003914. doi: 10.1371/journal.pmed.1003914 (PMC8903269; doi:10.1371/journal.pmed.1003914)
Supplement: S3 Table — (DOCX) [file pmed.1003914.s004.docx]

**S3 Table. Proposed indicators for sexual behavior/orientation stigma related to men who have sex with men**

| **Domain** | **Sub-Domain** | **Indicator** | **Included/**  **Excluded** | **Rationale for exclusion** |
| --- | --- | --- | --- | --- |
| Social norms and attitudes | Discriminatory attitudes towards men who have sex with men | Unclear | Excluded | No data currently available |
| Structural stigma | Criminalization of same-sex sexual acts | Existence of laws criminalizing consensual same-sex sexual acts | Included |  |
|  |  | Percentage who have been arrested because of having sex with men in the past 6 months | Excluded | Limited number of countries with data. No planned data collection |
|  | Non-discrimination laws | Existence of constitutional protections of discrimination or other non-discrimination provisions related to sexual orientation | Included |  |
|  |  | Existence of laws or other provisions that prohibit discrimination in employment based on sexual orientation | Included |  |
|  |  | Existence of a non-discrimination policy for students | Excluded | No data currently available |
| Violence | Recent experience of violence | Percentage of men who have sex with men who experienced physical and/or sexual violence in the last 12 months | Excluded | Limited number of countries with data. No planned data collection |
|  |  | Percentage of male gay students who have experienced physical, psychological, or sexual violence or bullying during the past 12 months | Excluded | No data currently available |
| Anticipated stigma | Anticipated stigma and discrimination experienced in accessing justice | Percentage of men who have sex with men who experienced physical and/or sexual violence in the last 12 months and who sought professional help or services and were refused services | Excluded | No data currently available |
|  |  | Percentage of men who have sex with men who experienced physical and/or sexual violence in the last 12 months and did not try to seek professional help or services because they were uncomfortable accessing services | Excluded | No data currently available |
|  | Anticipated stigma in healthcare settings | Percentage who avoided seeking healthcare in the past 6 months due to fear of stigma and discrimination | Excluded | Limited number of countries with data. No planned data collection |
| Experienced stigma | Experienced discrimination | Percentage of men who have sex with men who experienced discrimination or social exclusion in the last 6 months because they have sex with men | Excluded | Limited number of countries with data. No planned data collection |
| Internalized stigma | None | Percentage of men who have sex with men who report being ashamed to be men who have sex with men | Excluded | No data currently available |
|  | None | Percentage of men who have sex with men who report internalized sexual stigma (internalized homophobia) | Excluded | No data currently available |
