# Supplementary material for: Global assessment of existing HIV and key population stigma indicators: A data mapping exercise to inform country-level stigma measurement
Source: PLoS Med. 2022 Feb 22;19(2):e1003914. doi: 10.1371/journal.pmed.1003914 (PMC8903269; doi:10.1371/journal.pmed.1003914)
Supplement: S4 Table — (DOCX) [file pmed.1003914.s005.docx]

**S4 Table. Proposed indicators for sex work stigma**

| **Domain** | **Sub-Domain** | **Indicator** | **Included/**  **Excluded** | **Rationale for exclusion** |
| --- | --- | --- | --- | --- |
| Social norms and attitudes | Discriminatory attitudes towards sex workers | Unclear | Excluded | No data currently available |
| Structural stigma | Criminalization of sex work | Existence of any criminalization of sex work | Include |  |
|  |  | Percentage who have been arrested because of selling sex in the past 6 months | Exclude | Limited number of countries with data. No planned data collection |
|  | Non-discrimination laws | Existence of constitutional protections of discrimination based on occupation or other non-discrimination provisions specifying sex work | Include |  |
|  |  | Existence of laws or policies recognizing sex work as work. | Include |  |
| Violence | Recent experience of violence | Percentage of sex workers who experienced physical and/or sexual violence in the last 12 months | Exclude | Limited number of countries with data. No planned data collection |
|  |  | Percentage of sex workers who experienced abuse or threats from a client and/or were forced to have sex by a client in the last 6 months | Exclude | Limited number of countries with data. No planned data collection |
| Anticipated stigma | Anticipated stigma and discrimination experienced in accessing justice | Percentage of sex workers who experienced physical and/or sexual violence in the last 12 months and who sought professional help or services and were refused services | Exclude | No data currently available |
|  |  | Percentage of sex workers who experienced physical and/or sexual violence in the last 12 months and did not try to seek professional help or services because they were uncomfortable accessing services | Exclude | No data currently available |
|  | Anticipated stigma in healthcare settings | Percentage of sex workers who avoided seeking healthcare in the past 6 months due to fear of stigma and discrimination | Exclude | Limited number of countries with data. No planned data collection |
| Experienced stigma | Experienced discrimination | Percentage of sex workers who experienced discrimination or social exclusion in the last 6 months because they sell sex | Exclude | No data currently available |
| Internalized stigma | None | Percentage of sex workers who report being ashamed to sell sex | Exclude | No data currently available |
