# Supplementary material for: Global assessment of existing HIV and key population stigma indicators: A data mapping exercise to inform country-level stigma measurement
Source: PLoS Med. 2022 Feb 22;19(2):e1003914. doi: 10.1371/journal.pmed.1003914 (PMC8903269; doi:10.1371/journal.pmed.1003914)
Supplement: S5 Table — (DOCX) [file pmed.1003914.s006.docx]

**S5 Table. Proposed indicators for drug use stigma**

| **Domain** | **Sub-Domain** | **Indicator** | **Included/**  **Excluded** | **Rationale for exclusion** |
| --- | --- | --- | --- | --- |
| Social norms and attitudes | Discriminatory attitudes people who inject drugs | Unclear | Excluded | No data currently available |
| Structural stigma | Criminalization of drug use and/or possession | Existence of laws criminalizing drug use and/or possession for personal use | Included |  |
|  |  | Percentage who has been arrested because of injecting drugs in the past 6 months | Excluded | No data currently available |
|  | Non-discrimination laws | Existence of any specific anti-discrimination laws or other protective provisions that apply to people who use drugs | Included |  |
| Violence | Recent experience of violence | Percentage of people who inject drugs who experienced physical and/or sexual violence in the last 12 months | Excluded | No data currently available |
| Anticipated stigma | Anticipated stigma and discrimination experienced in accessing justice | Percentage of people who inject drugs who experienced physical and/or sexual violence in the last 12 months and who sought professional help or services and were refused services | Excluded | No data currently available |
|  |  | Percentage of people who inject drugs who experienced physical and/or sexual violence in the last 12 months and did not try to seek professional help or services because they were uncomfortable accessing services | Excluded | No data currently available |
|  | Anticipated stigma in healthcare settings | Percentage of people who inject drugs who avoided seeking healthcare in the past 6 months due to fear of stigma and discrimination | Excluded | Limited number of countries with data. No planned data collection |
| Experienced stigma | Experienced discrimination | Percentage of people who inject drugs who experienced discrimination or social exclusion in the last 6 months because they inject drugs | Excluded | No data currently available |
| Internalized stigma | None | Percentage of people who inject drugs who report being ashamed to be an injection drug user | Excluded | No data currently available |
