# Supplementary material for: Global assessment of existing HIV and key population stigma indicators: A data mapping exercise to inform country-level stigma measurement
Source: PLoS Med. 2022 Feb 22;19(2):e1003914. doi: 10.1371/journal.pmed.1003914 (PMC8903269; doi:10.1371/journal.pmed.1003914)
Supplement: Attachment — Submitted filename: Lyons_PLOS Stigma Indices response to reviewers final_FINAL.docx [file pmed.1003914.s008.docx]

**Response to reviewers**

Dear Editorial Team and Reviewers,

We are pleased to submit our revised manuscript, now entitled “Global assessment of existing HIV and key population stigma indicators: a data mapping exercise to inform country level stigma measurement”, to PloS Medicine. The original version of this manuscript was submitted to PloS Medicine under the title “Standardizing measures to track progress in eliminating HIV and key population stigma: A data mapping exercise”, and reviewed. We have incorporated the constructive feedback provided by the editor and reviewers into this updated version and believe the recommendations have much improved the manuscript. We sincerely appreciate the opportunity to respond to the comments provided and are happy to address any additional comments or feedback.

Below, we provide point by point responses to the reviewers' and editors' comments and detail the changes that we made in response. Please find both a clean and tracked changes version of the main article file included with this resubmission.

We have made revisions to the article’s format to align with the submission guidelines per editor instructions, including:

- Adding the label, title and legend for the figures in to the text of the manuscript.
- Uploading figures associated with our paper as individual TIF or EPS files with 300dpi resolution using the PACE digital diagnostic tool;
- Updating the data availability statement to align with PLOS Data Availability standards and ensuring that authors are not named as contacts for inquiries about access to study data in the data statement.

**Requests from the editors**

We notice that at least one author is named in the acknowledgements.

- Apologies for this mistake. We have removed any authors from the acknowledgements.

Please mention the dates of data acquisition in the abstract.

- We have added the following statement to the abstract: “Data extraction was conducted between August and November 2020.”

Please add a new final sentence to the "Methods and findings" subsection of your abstract, which should begin "Study limitations include ..." or similar, and should quote 2-3 of the study's main limitations.

- Thank you and we have added the following to the abstract: Study limitations include potential selection bias due to available data sources to the research team and other biases due to the exploratory nature of this process.

Please trim the "Conclusions" subsection of the abstract by about 50%.

- Thank you. We have reduced the length of the conclusions section of the abstract by 50%

After the abstract, please add a new and accessible "Author summary" section in non-identical prose. You may find it helpful to consult one or two recent research papers in PLOS Medicine to get a sense of the preferred style.

- Thank you and please find the author summary which has been added to the manuscript below:
  - Why was this study done?
    - Many people living with HIV and key populations, including gay men and other men who have sex with men, sex workers, people who use drugs, and transgender persons are affected by stigma.
    - Stigma is a barrier to HIV prevention, diagnostic, and treatment services and also negatively impacts the health and quality of life of those affected.
    - Although reducing stigma is an important pillar of the HIV response, there are currently no established summary measures to characterize the overall level of stigma within a country and track progress in addressing stigma over time.
    - The main purpose of this study was to review existing stigma data globally and assess which stigma indictaors have the potential to inform a summary measure of stigma at the country level.
  - What did the researchers do and find?
    - In this global mapping exercise, data for stigma indicators detailed in the following sources were reviewed and assessed: UNAIDS National Commitments and Policies Instrument database, Multiple Indicator Cluster Surveys, Demographic and Health Surveys; People Living with HIV Stigma Index surveys; HIV Key Populations Data Repository; Integrated Biological and Behavioral Surveys, and other databases.
    - Through this exercise, 24 HIV stigma indicators and 10 key population indicators were determined to have potential for use in the creation of valid summary measures of stigma at the country level.
  - What do these findings mean?
    - The results of this study highlight challenges in creating summary measures for stigma experienced by key populations or related to HIV based on existing global data sources. However, there is an opportunity to use participatory approaches alongside the existing data to create summary measures to describe and track stigma over time.

At line 95, for example, please remove spaces from within the reference call-outs (i.e., "... HIV pandemic [11,18].".

- Thank you and we have removed spaces within the reference call outs.

Please state early in the Methods section (main text) whether the study had a protocol or prespecified analysis plan.

- Thank you for this point; we have added the text below to the methods to clarify the use of a priori criteria. For further clarification, as an exploratory process we had a protocol and pre-specified criteria to inform the data mapping exercise, selection criteria, and assessment criteria. However, as this was a data mapping exercise, the specific cut-point for inclusion and categorizations for assessment were informed by the data obtained and extracted through this process. We have added the following text to the methods section.
  - This data mapping exercise is an exploratory process. The criteria used to identify, include, and assess indicators were established a priori. However, final decisions were informed by the data obtained as part of a larger plan to look at stigma metrics.

At line 239, please correct "indictors".

- Apologies for this error. We have corrected the spelling for this.

Please avoid "leveraged" in favour of "used", for example.

- Thank you, we have changed “leveraged” to “used” throughout the manuscript.

Throughout the text, please substitute "sex" for "gender" where appropriate.

- Thank you for raising this issue. We a have reviewed the use of sex and gender to ensure that the terms are used appropriately throughout the manuscript.

Please remove the information on funding from the end of the main text. In the event of publication, this information will appear in the article metadata via entries in the submission form.

- Thank you for clarifying. We have removed the funding information from the manuscript and have instead included this in the submission form.

Are references 3 & 19 missing journal names?

- Our apologies for this error. We have added the journal names for these references.

Please confirm that the maps used in the figures can be published under a CC BY licence.

- We confirm that the maps used in the figures can be published under a CC BY license.

Please add a completed checklist for the most appropriate reporting guideline, e.g., STROBE, labelled "S1_STROBE_Checklist" or similar and referred to as such in your Methods section (main text).

- Thank you very much for this. We have reviewed the STROBE Checklists and none of these seems to be designed for this type of exploratory data mapping study that examines data availability in secondary sources for a set of indicators. The STROBE checklists for cohort, case-control, and cross-sectional studies did not seem as though they would be appropriate for this data mapping exercise. We are very happy to fill a specific checklist if the editorial team and reviewers has a recommendation or suggestion for this.

In the checklist, please refer to individual items by section (e.g., "Methods") and paragraph number, not by line or page numbers as these generally change in the event of publication.

- Thank you and please see our response to the previous comment.

**Comments from academic editor:**

The reviewers have covered all the issues I have with this submission - the aim of this work is good, and addressing stigma in a methodologically sound manner is important for the reasons outlined in the paper. The reviewers all struggled with the presentation though, and all have provided valuable suggestions to clarify the aim, methods and results. Limitations need to be addressed in the Discussion too.

- Thank you very much for the comments and feedback. We have worked to address the reviewers’ concerns related to the presentation and hope that this revised manuscript has a clearer presentation of the methods and results. In addition, we have added the following limitations section to the discussion:

In addition to the challenges outlined in this discussion, there are several additional limitations to this data mapping exercise which should be considered. The objective of this study was to understand the stigma data that exists for calculating several stigma indicators that have potential use for informing a standardized stigma measure at the country level. As a data mapping exercise, this process was exploratory and therefore may be subject to bias. Although a priori criteria were established and are reported, final decisions on inclusion were based on the state of the data observed during this process. The research and authorship team includes a diverse group of stigma experts from across different academic institutions, multilateral agencies, community networks, and international organizations. This collaborative approach was taken to expand the access to existing stigma data, and to minimize selection bias. Our research team used publicly available data, supplemented by data obtained through collaborators, and directly through the research team where possible. This study was therefore unable to assess data that were not publicly available, or databases that were not yet available at the time of this data mapping exercise, but could have potentially been shared by institutions outside of this collaborative research team. Although systematic reviews of the literature may have informed which data had ever been collected, it would not have yielded the raw data needed to conduct this exercise (i.e., the data would not necessarily be accessible).

In the title, it would be helpful to clearly highlight the focus on Stigma (currently that sort of disappears a little amongst the other words).

- Thank you for the suggestion and we have revised the title to respond to your concern. The revied title is: “Global assessment of existing HIV and key population stigma indicators: a data mapping exercise to inform country level stigma measurement”

Given that stigma is likely to be culturally determined, and to vary across populations, possibly even within countries, there should be a clear discussion as to whether there should be a sort of 'basic' stigma data collection that would apply across regions/countries, with some additional summary data to apply to specific countries (which would enable within country tracking of stigma prevalence). Even within country there could be differences by population groups.

- Thank you for this comment and we completely agree. In the discussion section we have tried to highlight this concern and the need to consider this in the finalization of a summary measure with the following text:

There is heterogeneity in the experiences and burden of stigma within and across populations. An essential consideration, true in the creation of any summary measure for a complex construct, is that heterogeneity within countries will be masked in the process of distilling multiple indicators into one or even a few indices. This data mapping exercise demonstrated significantly more data characterizing HIV stigmas than those focused on sexual behavior or orientation, gender identity, sex work, or drug use-- challenging our ability to describe heterogeneity within the key population subgroups, such as age, residence, employment, etc. Discussions have emerged in this process to consider the development of a single summary measure of stigma for all key populations in order to clearly guide targeted HIV and human rights policy, advocacy and practice. Not only may this mask diverse experiences of stigma across these populations, but it may mask substantial differences in the relative state of stigma and progress towards its elimination. For instance, we found vast differences in the level of criminalization related to different behaviors associated with different key populations. However, creating separate indices may artificially segment experiences that are intersectional for individuals with HIV and membership of a key population. Intersectional stigma is the potentially compounded effect of stigmas among individuals with multiple identities which may be devalued by some in society. Unfortunately, the development of measures for intersectional stigma, are still in their nascency and would not be represented in this index. Additionally, the emerging field of microaggressions is an important area of ongoing research that aims to characterize and understand stigma processes, and although there is not yet consensus on how this fits within the stigma framework, there is likely great overlap with individual level experiences of stigmas. However, this is not represented in the current stigma indicators that were considered and whether and how to characterize such experiences at a broader socioecological level to understand stigma at a country level is an important area of future research. Given that summary measures may not be nuanced enough to appropriately represent intersectionality when combined, in balancing all considerations we recommend the creation of separate indices for HIV and each key population stigmas with ongoing consideration of how to examine intersectional influences on health. The addition of narratives from advocates and people with lived experience will be essential to consider in conjunction with any summary measure towards this purpose.

- Additional text included in the discussion to highlight this shared concern:

The process for creating summary measures to accurately describe and track country level stigma, while also accounting for heterogeneity and complexity is a challenge, yet not an insurmountable one. As described in conceptual frameworks depicting the processes of stigma, these relationships are complex and often do not occur in isolation [17]. In terms of understanding stigma, there is discussion about the appropriate point of measurement – whether it be the social determinants of stigmas (reflected for example in indicators such as acceptability of partner violence, demonstration of controlling behaviors, or having experienced intimate partner violence) or the experiences of stigmas as an outcome or endpoint (reflected for example in indicators such as having been socially excluded for living with HIV, having experienced HIV discrimination in healthcare, or having lost a source of income due to living with HIV). Continuing this discussion on the process and methods for stigma measurement at a country level will be an essential step for improving our ability to characterize stigma among and between populations and track country progress in addressing stigma. This should be done in conjunction with, rather than at the expense of, continuing work to measure and assess stigma at other socioecological levels, including the interpersonal and individual levels. Ultimately, measures of stigma at the country level can be used in combination with for instance, individual level stigma measures, in comparative research to help elucidate the complex interaction between structural stigma and other stigma manifestations.

**Comments from the reviewers**:

***** Reviewer #1: Statistical review**

The paper by Lyons and colleagues looks at the availability and utility of a range of indicators for measuring stigma relevant to the prevention, diagnosis, and treatment of HIV, derived from a number of international surveys and databases. This review considers the use of statistics in the paper.

Generally, I thought the paper was an interesting read. There is very little for me to review. The only statistics in the paper are those reported in the right-hand columns of Tables 2-6. These could be laid out a little neater. For example, looking at Table 1, indicators 3-6 are difficult to read, as each item goes over two lines, whereas indicators 7-9 have one item per line and are much clearer.

- Thank you very much for this feedback. We have tried to reformat slightly to make these neater.

When summarising an indicator that is the percentage of people with a particular response to a survey within a country, it might help to add "%", since this is the unit of measurement. E.g. indicator #1 in Table 1 would be shown as Mean (SD) = 44.4% (20.4%); Range: 5.7% - 81.4%. Note, I think "SD" is better than "sd".

Thank you and we have revised the presentation of all the summary measured based on your suggestion in reference to Table 2 indicator 1. We have also revised sd to SD and made additional formatting changes to improve the clarity of the presentation of the results.

In Table 1, indicator 14 ("Percentage of ever-married women whose husbands/partners demonstrated types of controlling behaviors"), we are given two measures, neither of which matches the definition. However, the second ("proportion of respondents reporting no controlling behaviors") is surely the opposite of what is needed, so could be converted by subtraction from 100%. Also, the summaries given are percentages, not proportions.

- Thank you and the original decision to include these two summary measures are based on how the data were reported in DHS. DHS reports the number who had 3 or more controlling behaviors or the number that had no controlling behaviors. As suggested by the reviewer, it would have been our preference to take the inverse of the second measure “percentage of respondents reporting no controlling behaviors”. However, this measure included a response option “don’t know” and the data had a high level of missingness. Given these limitations in the data we think it would not be appropriate to take the inverse of no controlling behaviors to match the wording of the proposed indicator.
- We recognize including both measures in this section may be confusing, and therefore we have now revised this to only include one measure. We selected the first measure “percentage of respondents reporting 3 or more controlling behaviors” to be included. This measure was selected because it has the same directionality of the proposed indicator and given the challenges with the second measure which could not be transformed to the inverse.
- We are also now reporting percentages.

For Table 1, indicator 15 ("Percentage of women age 15-49 who have experienced physical and/or sexual violence by an intimate partner in the past 12 months"), two sets of summaries are given, one of which matched the definition (available in 55 countries) and one that is slightly different (available in 53). To what extent do the 55 and 53 countries overlap, or are they different? What does the second definition add? Are the authors saying that it could be used as a proxy? If so, would it be better to report the total number of countries where a value for this indicator (by either definition) is available, and give summaries for the best estimate available, rather than reporting two?

- Thank you for highlighting this and we now have only included the measure and estimates for the indicator defined as physical or sexual violence. Given we are now reporting only 1 indicator and a single set of summary measure we are no longer reporting the second country availability.

In Table 1, indicator 11, there are two possible sources of information (Civil Society Report, or National Authority Report, though there could be both), but we are given a single set of summaries.

- Thank you and we updated the table to only include the reference for the summary provided.

For indicator 17, however, we get two sets of summaries, and it is not clear how many countries had data from at least one source. I think it would be better if we could be given a single set of summaries for each indicator, plus an explanation of how data from multiple sources are used to get a single value for each country (e.g. use one source in preference when both available, or use the average of the two).

- Thank you very much for highlighting this. We agree it would be preferable to have a single set of summaries for each indicator. However, there is not consensus on which source (civil society or national authority report) would provide better estimates. An advantage of the national authority report is that it may incorporate a broader knowledge base to inform their estimates of what policies are present or absent in a given country. However, an advantage of the civil society report is that they prioritize accountability and representation from community, and therefore may be more likely to report the presences of discriminatory policies with this perspective. Additionally, the preferred source may differ based on the specific indicator. We want to demonstrate that the use of these two sources does in fact result in different prevalence estimates. Given this, we have included both estimates where available, noting that the appropriate source for the estimates for each indicator needs to be determined.
- Thank you also for raising the point about potentially combining estimates. After considering this, we would not recommend combining these, and therefore we have removed the report of if one or the other reports were available – in order to not suggest the combining of these measures.

For the figures, there are a couple of minor observations. Figure 1 is slightly truncated compared to the panels of Figure 2 - it would look better if they were all the same. Also, the relevance of the red and grey dots (or are they asterisks?) was not clear. This also applies to Figure 2, though there are far fewer dots on these.

- Thank you and we have now made the figures consistent in truncation between figures 2 and 3 (previously 1 and 2) We have also removed the red dots for clarity.

***** Reviewer #2:**

This study is timely, and very important to ensure that we have a consolidated effort towards addressing stigma. I believe that a key barrier to addressing stigma also lies in the lack of consensus around how stigma can be conceptualized, operationalized, and measured. This study charts a way forward. I have some comments and suggestions below for your consideration:

Introduction

Overall, some additional references/citations as well as clarity around definitions of stigma would help strengthen the introduction:

- Thank you for this feedback. We agree and have addressed each of these in turn below.

1. Lines 77-78: Some citations would be useful to justify this claim

- Thank you, we agree and have added several citations.

2. Lines 79-85: These are great examples of stigma, however I think as the manuscript is attempting to review measures of stigma, it would be useful for the authors to clarify how anticipated, perceived, internalized, or enacted stigma differ. In fact, enacted (or experienced) stigma often refers to discrimination in most definitions. Some brief definitions for each term introduced and how they might overlap or be distinguished from each other would be useful.

- Thank you for this comment and we have added definitions of anticipated, perceived, internalized, and enacted stigmas to the introduction to clarify how each if distinct. The added text is:
  - Anticipated stigmas refer to the expectation of bias perpetrated by others. Perceived stigmas refer to felt experiences of stigma or discrimination and the perception of bias as understood by a person living with a stigmatized identity. Internalized, or self-stigma, is the adoption of negative feelings or devaluing of oneself on account of a stigmatized identity. Enacted, or experienced stigma, is the perpetration of mistreatment or discriminatory acts by someone.

3. Line 100: It would be useful for the reader to define what intersectional stigma refers to.

- Thank you and based on feedback from another reviewer we have moved first mention of intersectionality into the discussion section and included a definition when the term is introduced. The new text is:

Intersectional stigma is the potentially compounded effect of stigmas among individuals with multiple identities which may be devalued and stigmatized by some in society.

4. Lines 105-112: A reference/citation for this would be useful for the reader.

- We agree and have added a reference here.

Methods

Overall, the methods section had a thick description of the processes involved in the study. Just a few comments and suggestions:

1. Lines 134-135: I think there is sufficient information here on the backgrounds of the participants, but it would be useful to describe if there were any observable differences between the 804 who registered, versus those who eventually participated (if available).

- Thank you, and this is a great point, Unfortunately, we do not have descriptive data on the characteristics of people who registered but did not participate and agree that this information would be useful. In an effort to describe those individuals who participated, we have now included a table in the supplementary material with the distribution of geographic regions and sectors of work. This is included as S1 Table. Characteristics of participants in E-consultation.

2. Lines 165-166: Would reverse coding refer to index items where questions were reverse coded? Just a brief explanation would help contextualize this better.

- Reverse coding was done to ensure measures were representing the same direction or endorsement such as “no physical or sexual violence” to “any physical or sexual violence” We have revised the text in the manuscript to:

After data were extracted from these sources, further cleaning was conducted. This included reverse coding to ensure consistent direction of endorsement where necessary, and the identification of outliers.

3. Line 166: Which summary statistics were calculated for all indicators? It would be useful to mention them here.

- Thank you and we have added the following text to the methods section:

Descriptive statistics were calculated for all indicators with existing data, including, number, proportion, as well as mean and standard deviation when appropriate.

4. Lines 167-168: "Indicators were then selected based on their potential for creating a summary measure, also known as an index." - It would be useful to briefly describe how 'potential' was assessed and what processes went into this decision-making stage. Upon further reading throughout the rest of the paragraph, it seems like these were part of the processes to assess 'potential', but it isn't clear if these were done over and above an initial selection process (as described in the first sentence of the paragraph). Some clarity would help,

- We appreciate you highlighting that this was not clear. We have revised the opening sentence of this paragraph to the following:

Indicators were then selected based on a set of a priori criteria, outlined below, for their potential for creating a summary measure, also known as an index.

Results

Overall, the results were detailed and easy to follow. Just some suggestions for the authors' consideration:

1. Line 210: I generally would be cautious of grouping anticipated and experienced stigma together as these are unrelated concepts (i.e. anticipated stigma can be high in the absence of experienced stigma). Some commentary on this in the discussion / limitations could be useful.

- Thank you and appreciate this comment and agree about the challenges that may arise in grouping anticipated and enacted stigmas into the same domain. The decision of the domain was made during the expert consultation led by UNAIDS. The justification of this was that anticipated and enacted are linked in their influence on each other – as in anticipated stigma is often linked to an experience of enacted stigma. Given identification of fewer possible anticipated stigma indicators, grouping these together allowed for consideration of both. However, we fully appreciate that these types of stigmas are distinct and have revisited this decision. During this revision, we have decided to separate these domains into “experienced stigma” and “anticipated stigma”. You will see this change reflected in the manuscript and tables.

2. Lines 271-273: The figures are a great way to illustrate the spread of countries by data availability - however a data table would be useful as supplemental material as well.

- Thank you and we agree a table would be very useful. We currently have 6 tables and 3 figures in the main results as well as 6 supplementary tables and worry about including additional tables with duplicate information. We appreciate this, and if the reviewers and editorial teams prefer the table format instead of the figures, we are happy to make this change.

Discussion

The discussion is robust and highlights many considerations that the results of this study surface. Some suggestions for the authors' consideration below:

1. Lines 301-306: "These indicators provide some ability for beginning to explore if and how complex experiences of stigma may be able to be summarized to elucidate the state of stigma within a country and assess change going forward." - Can the authors suggest some qualities of these indicators? These would be useful to help guide future participatory approaches to the creation of summary measures.

- Thank you and we have revised this statement to the following to provide more detail on what was considered in this mapping exercise to demonstrate potential utility. Please see the revised text here:

Given geographic representation, repeated implementation, and quality, these indicators provide some ability to explore if and how complex experiences of stigma may be able to be summarized to elucidate the state of stigma within a country and assess change going forward.

2. I believe that one key shortcoming in our general discussions around stigma is the lack of consensus around the definitions of stigma. This is evident in the groups of anticipated and experienced stigma - if it is meant to capture individual level stigma, then it should also include internalized stigma. I think future efforts need to involve interdisciplinary academic teams (social psychology, sociology, psychology, health behavior scientists, public health professionals etc.) that have diverged in the ways that they have defined stigma to find consensus over what works for summary indices. Some synonyms include 'felt/perceived' stigma, 'internalized/self' stigma, 'experienced/enacted stigma or discrimination'; some scholars have also argued for stigma such as 'project' stigma, which refers to a form of resistance against stigma.

- Thank you and we completely agree with this statement and perspective. We appreciate the lack of consensus around the definitions of stigma and terminology used. In response, we have provided definitions of the different types of stigma at the beginning of the article, and we have tried to incorporate alternative terminology used into these definitions. Please see this text below:

Stigma is a social process in which an individual or group is linked to a negative stereotype or misconception, resulting in adverse experiences, loss of social status, and limited opportunities. Stigma may occur at the individual, interpersonal, community, and structural levels and can be experienced as anticipated, perceived, internalized, or enacted. Anticipated stigmas refer to the expectation of bias perpetrated by others. Perceived stigmas refer to felt experiences of stigma or discrimination and the perception of bias as understood by a person living with a stigmatized identity. Internalized, or self-stigma, is the adoption of negative feelings or devaluing of oneself on account of a stigmatized identity. Enacted, or experienced stigma, is the perpetration of mistreatment or discriminatory acts by someone. Discrimination, part of the social process of stigma, includes any distinction, exclusion or restriction made to human rights and fundamental freedoms, in the political, economic, social, cultural, civil or any other field. Discrimination can be institutionalized through existing laws, policies or practices that negatively impact people living with HIV or key populations. Although the conceptualization of discrimination in relation to stigma varies, here we consider enacted stigma to be inclusive of discrimination.

- Please also mote that based on the feedback from another reviewer we have no separated the domain “anticipated and experienced stigma” into two domains “anticipated stigma” and “experienced stigma”

3. Related to point #2 above, an emerging field where indices are being developed include HIV microaggressions, or microaggressions experienced by key populations. How do microaggressions fit into the overall stigma framework? Overall, I think a paragraph dedicated to addressing the lack of consensus for now, and warning about the divergence in such conceptualizations of stigma, would be important.

- Thank you for highlighting the overlap of this work with the emerging field of microaggressions, as this is an incredibly important body of work. We have added the following to the discussion in recognition of this:

Additionally, the emerging field of microaggressions is an important area of ongoing research that aims to characterize and understand stigma processes, and although there is not yet consensus on how this fits within the stigma framework, there is likely great overlap with individual level experiences of stigmas. However, this is not represented in the current stigma indicators that were considered and whether and how to characterize such experiences at a broader socioecological level to understand stigma at a country level is an important area of future research.

***** Reviewer #3:**

GENERAL COMMENTS

The topic the authors address in this manuscript is globally very relevant across different settings. It is very challenging to find a summary measure to routinely quantify the stigma burden related to HIV status or key population membership at national level, which would enable tracking this burden over time in individual countries and comparing it among different countries in a more accessible way. The authors included a number of stakeholders in this exercise, explored an extensive number of data sources and provided an interesting insight into the current status and their view of the way forward.

However, the lack of clear and detailed description of methods hampers overall clarity of other sections of this work, and it is therefore difficult to judge whether this paper is suitable for publication in Plos Medicine. Several different aims and goals are presented throughout the paper, which is confusing. My understanding is that the authors aimed to review available indicators and data for the period from 2000-2020, track progress over that specific period in individual countries worldwide (retrospectively), summarize the findings in this paper, and them, at some later point, create a summary measure to be used at country level. However, the authors' focus seems to be on the summary measure in the Title/Abstract/Introduction/Discussion, whereas in the Methods/Results they relatively briefly describe their methodological approach. The methods they used seem to have some limitations, substantial bias may have been introduced, a more systematic approach may improve the technical quality. It may be useful to present methods in a clearer (step-by-step) way, as it may also be helpful for researchers working on stigma in other areas (outside of HIV) to use it as a roadmap for similar endeavors in their areas in the future.

- We are very appreciative of this review. We have worked to address your comments which are outlined in a point-by-point response below to each of the comments provided and we feel this has substantially strengthened the paper. Thank you for your detailed comments, critique and feedback.

SPECIFIC COMMENTS (*=major)

Title and Abstract:

1. The Abstract seems quite long, a shorter, clearer version may be more appropriate to engage the reader

- Thank you and we have revised the abstract, in particular shortening the conclusion in response to your comment below and the feedback from the editor.

2. Conclusions in the abstract seem disproportionally long

- Thank you and we have revised and shortened the conclusions section of the abstract.

3. The title and the abstract are not fully aligned with the content of the main text (e.g. timeframe, inclusion of all countries irrespective of their geographical region/income category etc.)

- Thank you, we agree that a more descriptive title better aligned with the objective of this paper is warranted. We have revised the title of the manuscript in an effort to better represent this work to: “Global assessment of existing HIV and key population stigma indicators: a data mapping exercise to inform country level stigma measurement.”

4. The way the aim of this work is presented in the main text is confusing, the aims in the main text and in the abstract seem inconsistent.

- Thank you for this feedback and we have revised the language in the main text and revised the abstract to be in better alignment.

Introduction:

1. The first sentence, L64 is unclear, please rewrite

- Thank you and we have revised the sentence to the following:

Despite significant advancements in HIV prevention, early detection, and treatment, nearly 40 million people are living with HIV around the world and there were an estimated 1.5 million new infections in 2020.

2. Suggest using standard abbreviations throughout the text for all key populations, and not only for MSM. Some researchers argue that such abbreviations are dehumanizing, and if this is the reason for authors not to use them, the abbreviations should also be omitted for MSM for consistency. This applies to the Abstract too.

- Thank you very much for this comment. The use abbreviations was a large topic of discussion during the development of this manuscript for the exact considerations you describe. We do think that the use of some abbreviations for some populations can have a dehumanizing element. To be consistent across populations, we now spell out men who have sex with men instead of using the abbreviation throughout.

3. Please add reference to support the statement in the first paragraph L77-78.

- Thank you and we have added several references to support this statement.

4. Please provide a definition of stigma in the second paragraph. I also suggest a sentence clarifying the terms "measure", "indicator", index"

- In response to this comment and that of reviewer #2, we have included the following definition of stigma in the second paragraph:

Stigma is a social process, in which an individual or group is linked to a negative stereotype or misconception, often resulting in adverse experiences, loss of social status, and limited opportunities.

- We have also added the following text in the introduction:

For the purposes of this work, we consider an indicator to be a single item measuring an aspect of stigma, and an index is a composite of indicators to generate a summary or average value representing a broader stigma construct.

5.L103, 104 "A summary measure will allow…" the wording does not reflect the uncertainty, as the measure has not yet been developed; suggest "may allow" or similar; as similar content appears in the last paragraph of this section, consider removing

- Thank you for pointing this out and we have revised the statement to reflect uncertainty:

A summary measure to quantify progress towards achieving zero discrimination may allow for easy comparisons within and across countries, and possibly improved accountability for progress.

6.L105-106 "achieve the achieving", please check and rewrite. There are several other typos throughout the text, please check and correct.

- Thank you and we have corrected this error and thoroughly edited the manuscript in response to this feedback as well as that of Reviewer #4.

7.* Challenges with the HIV indicators in terms of geographical and temporal consistency are not limited to stigma and discrimination indicators- as this is a journal read by a broad audience, in order not to mislead the readers, authors should present a more balanced perspective of the general challenges with the HIV and key-populations related indicators and M&E. The authors mention progress with the stigma indicators (L97), and that there are still challenges with intersectional stigma and a summary measure - this should be rewritten so that it is clearer for the reader what the main point of the paper actually is, what is the knowledge gap that they are addressing in this paper

- Thank you for highlighting this and we really appreciate this perspective. We agree that challenges in HIV indicators in terms of geographical and temporal consistency are not limited to stigma and discrimination indicators, and we have worked to make sure the language of this manuscript does not suggest measurement challenges are limited to stigma. However, we worry that discussing other challenges of HIV measurement may distract the reader from the specific goal and scope of this work. In this revised manuscript we have tried to be more precise in our aims and scope and hope that this comes through clearly. We welcome continued feedback in terms of making sure this manuscript is accessible to a broader audience. We have also added the following text to the introduction: Stigma measurement has unique challenges given it is a latent construct, and a social process which acts across multiple levels, and a phenomenon that has often been thought of as too complex to measure.
- We have moved the narrative on intersectional stigma measurement from the introduction to the discussion to not distract from the main gaps of current summary measure development, and the objectives of this study. We hope that this section of the introduction in now clearer for the reader to follow.

8. *In general, there seems to be some repetition throughout this section, it would be easier to follow if it was shorter and some parts, especially those similar to the ones appearing in the Discussion section again removed.

- We really appreciate this feedback and have revised the introduction and discussion to remove repetition and to be overall, more concise.

Methods:

1. L124 "UNAIDS" seems vague, please specify which Team/Office or similar.

- Thank you and we have updated the text to clarify this process was led by the UNAIDS Strategic Information Department. The revised text is: UNAIDS Strategic Information Department led an initial drafting of possible domains, sub-domains, and indicators to be considered for inclusion in summary measures of stigma affecting people living with HIV and four key populations: men who have sex with men, sex workers, people who inject drugs, and transgender people

2.* L127 please add reference for GAM. GAM indicators and other established indicators (WHO SI guideline) should be mentioned explicitly in the Introduction, where there's a statement on progress in developing measures (L97)

- Thank you and we have added a reference to GAM, and have also explicitly included the following statement in the introduction with references to GAM and WHO SI guidelines: Specifically, progress has been made through the Global AIDS Monitoring indicators and the WHO Strategic Information guidelines.

3.* Several different aims and goals presented in the Introduction section L105-120 and again in the Methods L127-129, this is confusing, please rewrite and present the aims in one paragraph and align the text in the Abstract.

- Thank you for this helpful feedback and we have revised the main text and abstract accordingly.

4. Suggest to present the whole process (step by step) described in Methods in a flowchart, the text alone is difficult to follow

- Thank you for this feedback and we have now included a flow chart as Figure 1 to outline the methods.

5.* The way the E-consultations are described in L131-140 is somewhat confusing - it is important for the readers to be able to judge for themselves how balanced and representative the group participating in the e-consultations was, there are no details on what proportion of participants were from which region, stakeholder group etc., a disbalance may have caused bias. If there is a separate report on this process it should be referenced, so that the readers can access it and judge for themselves.

- Thank you for this comment. In response, we have included a supplementary table (S1 Table. Characteristics of participants in E-consultation) to provide detailed information on participants.

6. Suggest using bullet points for data sources and adding references to IBBS Studies which were included if they were published or references to their protocols.

- Thank you and we have now included references to manuscripts which describe the implementation of the IBBS studies.

7.* IBBS studies implemented by authors' institution and Sub-Saharan Africa only included, which may have introduced bias, the results may therefore not be generalizable in the global context.

- Thank you and we completely agree that the results from the IBBS studies in Sub-Saharan Africa would not be generalizable to the global context. We would like to highlight, that this data mapping exercise does not attempt to generalize estimates, rather the aim was to use the data that were available to the study team to understand what measure could be used to inform a summary measure. It was determined that none of the measures from the IBBS studies could be used as these were only available for a small set of countries.

8.* The authors did not perform a systematic review of published literature and have not extracted data in a systematic way, with a pre-published protocol available for independent verification, IBBS studies in some countries may have been missed. This is a major methodological limitation, as indicators were later excluded based on the number of countries for which data were available (L169).

- Thank you very much for this important comment. We have now included text to provide more details on the databases searched for work and the outlined the limitations.
  - This team did not conduct a systematic review of literature, as the objective was the review raw data available. We did however review a database which developed from data obtained in a comprehensive systematic review of HIV-related data among key populations. The protocol for this systematic review is registered in the PROPSERO database and is in accordance with the guidelines specified in the Preferred Reporting Items for Systematic Reviews and Meta-analyses statement for protocols (PROPSERO RD42016047259). We have included the revised text in the methods section to clarify this and have providing a citation to the original systematic review: The Key Populations Data Repository, which is a database was developed from a comprehensive review of all available data for key populations, including burden and risk of HIV, prevalence, incidence, prevention indicators and treatment cascades, population size estimates, experienced violence, and engagement with healthcare systems was reviewed.
  - We have also worked to clarify the objective of this work to address this comment and have also incorporated some language to clarify this and added the to limitations. We have added the following text to the discussion: In addition to the challenges outlined in this discussion, there are several additional limitations to this data mapping exercise which should be considered. The objective of this study was to understand the existing stigma data and its potential use for informing a standardized stigma measure. Given this, the research team leveraged publicly available data, data obtained through collaborators, and data available directly through the research team. Therefore, this study was unable to assess data that were not publicly available but could have potentially been shared by institutions outside of this collaborative research team. Therefore, the results may be subject to selection bias. Although systematic reviews of literature may have informed which data had ever been collected, it would not have yielded the raw data needed to conduct this exercise. Additionally, as a data mapping exercise, this process is exploratory in nature and therefore may be subject to bias. Although a priori criteria were established, cut points for inclusion were based on the state of the data observed during this process. The research and authorship team includes a diverse group of stigma experts from across different academic institutions, multilateral agencies, community networks, and international organizations. This collaborative approach was taken to expand the access to existing stigma data, and to minimize selection bias of the selection and assessment of data.

Results:

1.A flowchart with included indicators at every step and excluded indicators (with reasons for exclusion) would add clarity and be easier to follow

- Thank you and is described in Table 1: Summary of stigma indicator mapping identification, extraction, selection, and assessment, which attempts to describe each step of the selection and assessment process and how many indictaors were excluded at each phase of this exercise.

2. *Many decisions on exclusion and inclusion of indicators also seem to have been done arbitrary and not in a consistent way (e.g. L185 "Indicators determined to be redundant were removed from consideration" vs L251 "Based on decisions to create separate summary measures for key populations … we did not drop any indicators from further consideration based on potential redundancy.")

- Thank you very much for highlighting this. We have revised the statement in the methods to clarify that we would consider removal of redundant indicators. The updated text in the methods is: Indicators determined to be redundant were considered for removal.
- Thank you also for highlighting that the presentation of our results in this section were not presented clearly. We have revised this section to provide more detail and explanation of the results and rationale for the decision for inclusion and exclusion. Please find the revised section below:

Several sets of indicators were combined due to how the data were reported. Among indicators for sexual minority stigma related to men who have sex with men, the indicators “Existence of constitutional protections of discrimination or other non-discrimination provisions related to sexual orientation” and “Existence of laws or other provisions that prohibit discrimination in employment based on sexual orientation” were assessed via items that shared a “No” response option and are therefore collapsed into a single measure. Among indicators for sex work stigma, “Existence of constitutional protections of discrimination based on occupation or other non-discrimination provisions specifying sex work” and “Existence of laws or policies recognizing sex work as work” are collapsed into a single measure as these items were also collected with a shared “No” response option. Similarly, two of the five indicators for gender identity stigma with data available from more than just a few countries had to be combined due to how the data was reported through the NCPI (i.e. the questionnaire was structured so that indication of presence or absence of laws or policies assessed in these indicators shared a “None of these policies” category). These indicators were collapsed into a single measure.

The indicator “Percentage of people living with HIV who have lost a source of income or job because of their HIV status in the past 12 months” had a Pearson correlation coefficient of 0.74 with “Percentage of people living with HIV who have been refused employment or a work opportunity because of their HIV status in the past 12 months,” suggesting that inclusion of both of these indicators may not be necessary due to potential redundancy. We recommend retaining the former and excluding the latter given the former has data available for one additional country. For future assessment, PLHIV Stigma Index 2.0 index combines these two indicators which resolves this issue going forward [44]. These same indicators were correlated with “Percentage of people living with HIV who experienced social exclusion in the last 12 months due to their HIV status” (rho=0.82 and 0.84, respectively). “Feeling shame and guilt” and “Avoiding healthcare out of fear of discrimination” were also correlated at rho= 0.63. Although these indicators are correlated, they are not from the same sub-domain and therefore it is not recommended that they are collapsed or removed at this stage.

Among key population indicators, the sexual minority and gender identity “Discrimination and employment protection policies” indicator were correlated at a rho=0.68, however given these are different key populations (men who have sex with men; transgender persons) these indicators were retained at this stage. The sexual minority “Discrimination protections” indicator was correlated at a rho=0.54 with the “Provisions allowing for name change indicator”, however was not combined given the rho<0.60. Although some indicators showed correlation, no indicators were removed at for redundancy at this stage.

3. L273 "the greatest proportion of countries with available data for HIV and key population indicators is in sub-Saharan Africa." is not a surprising finding, as many studies were done there, however, due to the above listed limitations in methodology, some countries in other regions may have been missed. E.g. a systematic review by Fitzgerald-Husek et al. (Ref. 19) covered papers that measured stigma affecting MSM and SW from 2004 to 2014, most of which originated from high income countries (mostly North America), which seems not to be in line with the above statement.

- Thank you for highlighting this important point and we have removed this statement from the results and discussed selection bias in the limitations section.

4. Overall, the results are somewhat difficult to follow, please consider visual presentation instead of narrative

- Thank you and we have updated table 1 to better outline the results in a step-by-step process.

Discussion:

1.*This section is difficult to comment as the Methods and Results sections are unclear. The authors, however, do not include their view of the limitations of this study, which would be an important bit.

- Thank you and we have worked to clarify the methods and results section based on the feedback provided from the reviewers. We hope that the methodological process and results are easier to follow. We have also added the following limitations section to the discussion:

In addition to the challenges outlined in this discussion, there are several additional limitations to this data mapping exercise which should be considered. The objective of this study was to understand the stigma data that exists for calculating several stigma indicators that have potential use for informing a standardized stigma measure at the country level. As a data mapping exercise, this process was exploratory and therefore may be subject to bias. Although a priori criteria were established and are reported, final decisions on inclusion were based on the state of the data observed during this process. The research and authorship team includes a diverse group of stigma experts from across different academic institutions, multilateral agencies, community networks, and international organizations. This collaborative approach was taken to expand the access to existing stigma data, and to minimize selection bias. Our research team used publicly available data, supplemented by data obtained through collaborators, and directly through the research team where possible. This study was therefore unable to assess data that were not publicly available, or databases that were not yet available at the time of this data mapping exercise, but could have potentially been shared by institutions outside of this collaborative research team. Although systematic reviews of the literature may have informed which data had ever been collected, it would not have yielded the raw data needed to conduct this exercise (i.e., the data would not necessarily be accessible).

2. It may be useful to present methods in a clearer (step-by-step) way, as it may be helpful for researchers working on stigma in other areas (outside of HIV) to use it as a roadmap for similar endeavors in their areas in the future and provide comment on relevance for areas outside of HIV too in the Discussion section.

- Thank you for this feedback and we have now included a flow chart as Figure 1 to outline the methods so that the process may be clearer and that others may use this process in other areas of work.

Additional comments:

1.Table 2: I suggest adding a column with data sources where individual indicators were identified, and if there are commonly used or validated indicators in place, which indicators (e.g. GAM, WHO SI etc.) 2.Tables 2-6 Unclear what "Descriptive statistics" actually describes, this should be specified, abbreviation explanations should be added to footnotes of the tables 3. Fig 1 and 2 Footnote-suggest "more" instead of "greater"

- Thank you and we have included a column in tables 2-6 with the data sources for each of the indicators.
- We have revised the footnote for the figures to state ‘more’ instead of ‘greater’.

***** Reviewer #4:**

The article presents the summary of an interesting exercise that would address a clear need. The nuances of stigma within/between communities and populations would make codifying community-population level indicators (esp at the country-level) challenging, if not nearly impossible. This is indeed true when you factor the various causes of stigma, and the attitudes, policy, and cultural norms that fuel stigma. The scientific rationale for this is clear and established, the methods were sound. There are some grammatical/syntax revisions that should be made. Important consideration for future inquiries about developing summary indicators based on population-level data.

- We appreciate this critique and have reviewed the manuscript and made revisions to improve the grammar and syntax used.

***** Reviewer #5:**

First of all, congratulations for your comprehensive works and great efforts. The manuscript is clearly presented and it was accurately worked by current literatures of HIV to fulfill the study objectives. Sufficient details of methods and analysis are provided and applicable for its interpretation. The findings are described well and the conclusions are drawn adequately supported by the results.

For more clarification, reviewer' comments are provided as follows:

In methodology, the steps to combine different data for data mapping should be included.

- Thank you for this point. We have added the following text to provide more detail on the combination of data sources:

With the extracted data, data aggregated to the country level was combined to create country level database. Importantly, not all estimates in this dataset are national representative.

The number of countries extracted from each database should be mentioned for more detailed methodology.

- Thank you for this suggestion and the number of countries extracted varied by indicator in addition to database. Therefore, we have presented this by indicator to provide a more detailed picture in tables 2-6.

It is interested to know how the denominator is defined for the proportion of countries for which data were available.

- Thank you and we agree this was unclear and needed. We have worked to clarify the denominator in the presentation of the results, and also revised the following statement to clarify that the denominator for considering geographic spread of indicators is the number of UN member states:

The proportion of countries for which data were available for the indicator was determined to be ‘sufficient’ if it was 50% or more of UN member states.

(Line 190) It may be better to mention about the reason of setting the cut off of 50% to define sufficient and limited indicators.

- Thank you and this was simply based on having data available for the majority of UN member states, and we have revised the text to make this rationale clear. Notably, data availability for indicators did not often fall in close proximity to this cut-off, making our recommendations not particularly sensitive to the exact cut off used. We have revised the text in the methods section to:

The cut of 50% was meant to demonstrate data availability for the majority of UN member states (countries).

(Line 192 to 194) For "sufficient" available data within the country, is there any consideration of each domain in addition to overall indicators?

- Thank you for this question. We presented our results by domain in tables 2-6, to allow the reader to understand these indicators in the context of the domain in which they were considered to belong based on recommendations by the task force team., However, we did not include or exclude indictaors based on their domain, especially given the low number of indicators per domain the currently methodology yielded.

In data availability and quality, rationale for exclusion of Violence; Stigma and discrimination; and Internalized stigma for key population should be mentioned like explanation about no consideration of the social Norms and Attitudes domain.

- Thank you and we have updated the text to clarify that these domains were not excluded on some conceptual basis or consideration of importance, but on account of what data was available. Please see the following:

Outside of HIV stigma, no data were identified for indicators within the domains of social norms and attitudes and internalized stigma. Additionally, no indictaors within the domain of stigma and discrimination had sufficient data across countries or planned data collection for measurement over time for key populations. Therefore, these domains could not be considered for stigmas related to key populations after completing data mapping at this time.

- We have also updated the following text in the discussion section:
  - No publicly available data sources for appropriately assessing indicators within the domains of social norms and attitudes, or internalized stigma for key populations were identified, and therefore these domains had to be removed from key population stigma summary measures at present.

(Line 233 to 235) It is not clear that although it is mentioned "Feeling shame and guilt" and "Avoiding healthcare out of fear of discrimination" were not dropped to be consistent for key population indices (Line 247), these indicators were excluded in table of final indicators for key population because of no data availability. So, it will be better to give other explanation.

- Thank you very much for highlighting this. We have revised the statement in the methods to clarify that we would consider removal of redundant indicators. The updated text in the methods is:

Indicators determined to be largely redundant based on high intercorrelation with other indicators were considered for removal.

- We have also revised the statement in the results to better reflect these decisions. Please see our response to a similar comment from reviewer 3.

It should be mentioned the total number of countries as denominator about availability across countries for HIV stigma and key population stigma. (Line 264) For figure 1, it may be better to show all UN member states regardless of "sufficient" or "limited" number of HIV stigma indicator like figure 2.

- Thank you and we have revised this text to the following:

For the 24 remaining indicators of HIV stigma, 11 (45.8%) were identified as having data available in more than half of the 193 UN member states.

The color legend should be included for clear description of the map for all figures.

- We have now included a color legend on the maps.

Of the 24 potential HIV stigma indicators, it was found that there were 20 indicators that have potential for use in tracking change in stigma over time that shown in the table 2. So please clarify this number that was mentioned as 19 in Line 287.

- Thank you for highlighting this. In Table 2, 4 indicators were determined to have ‘limited’ ability to assess change over time, and 1 indicator was determined to not be able address change over time (‘cannot’). I think the confusion may have arisen from the reporting of indicators in the “Discrimination experienced in employment” domain as indictaors in this domain is combined for future data collection, and therefore treated as a single indicator moving forward. We have tried to clarify this in the results section and the table.

In Line 288, 11 HIV stigma indicators which had data available in more than half of countries were good to mentioned for clear presentation to the readers. It is also better to explain how half of countries is calculated.

- Thank you and in response to this and other comments from reviewers, we have included how half the countries is calculated in the methods section and have tried to reiterate this in the results for clarity (please see more detail in our other responses related to this point).

Conclusion in the body of manuscript and in the abstract should be similar.

- Thank you, and we have revised the abstract to better align with the main text of the manuscript.

In line 366, what does "n" mean between limitations and the current data?

Thank you for noting this grammatic error, which we have now corrected.

It may be better to explain how the domain and subdomain of stigma were considered or defined and how the indicators were categorized for each sub-domain. The literatures based for this categorization should be cited.

- Thank you very much for this suggestion. The proposed domains were discussed during the e-consultation, but the task committee did not create specific, agreed upon definitions. However, we have defined terms that may have needed clarification throughout this revised manuscript in response to reviewers’ comments.
