# Supplementary material for: Global assessment of existing HIV and key population stigma indicators: A data mapping exercise to inform country-level stigma measurement
Source: PLoS Med. 2022 Feb 22;19(2):e1003914. doi: 10.1371/journal.pmed.1003914 (PMC8903269; doi:10.1371/journal.pmed.1003914)
Supplement: Attachment — Submitted filename: Response_S3_2022 1 13.docx [file pmed.1003914.s009.docx]

**Response to reviewers**

Dear Editorial Team and Reviewers,

We are pleased to submit our revised manuscript, entitled “Global assessment of existing HIV and key population stigma indicators: a data mapping exercise to inform country level stigma measurement” (PMEDICINE-D-21-02404R3), to PloS Medicine. We have incorporated the remaining feedback provided by the editor and reviewers into this updated version. We sincerely appreciate the opportunity to respond to the comments provided and are happy to address any additional comments or feedback.

Below, we provide point by point responses to the reviewers' and editors' comments and detail the changes that we made in response. Please find both a clean and tracked changes version of the main article file included with this resubmission.

**Requests from Editors:**

In the data statement (submission form), please use the form "... data are ..." throughout. Please check through the main text for consistency on this point too (e.g., "... data exist" at line 149).

- Thank you and we have revised the submission form, line 149, and text throughout to align with this feedback (line 440).

We suggest adding a few words to the abstract to mention the e-consultation and the number of candidate indicators found thereby.

- Thank you and we have added the following to the abstract: UNAIDS Strategic Information Department led an initial drafting of possible domains, sub-domains, and indicators, and a 3-week e-consultation was held to provide feedback. From the e-consultation, 44 indicators were proposed for HIV stigma; 14 for sexual minority stigma (including sexual behavior or orientation) related to men who have sex with men; 12 for sex work stigma; 10 for drug use stigma; and 17 gender identity stigma related to transgender persons

At line 84, rather than "nearly 40 million" please quote an estimate from the relevant reference.

- Thank you and we changed this to the specific number: 37.7 million.

Thank you for your comments about the design of the study. We ask you to include the study protocol as an attachment, labelled "S1_Protocol" or similar and referred to as such in the Methods section, so that readers can judge the extent to which the findings were data-driven.

- Thank you and we have added the study protocol to the supplementary files.

We felt that the wording of the first paragraph of the Discussion section (main text) was somewhat more downbeat than the discussion in the abstract and final paragraph of the paper, and you may wish to revisit this component.

- Thank you and we have made some revisions to better align the first paragraph of the discussion with the final paragraph of the paper and abstract.

At line 369, we suggest amending the text to "In 2022, ..." or similar.

- Thank you and we have made this change.

Should that be "for key populations" at line 506?

- Thank you for pointing this out, and we have made this change.

At line 533, should that be "were" rather than "include"?

- Thank you and we have made this change.

In the reference list, please amend the typo in reference 2.

- Thank you and we have made this change.

Again in reference 18 there is a typo, and it appears that full access details need to be added to this citation.

- Thank you and we have made this change, which now appears as reference 20.

Can a report number or URL be added to reference 24?

- Thank you and we have added this.

Please check reference 38 for typos.

- Thank you and we have updated this.

Where available, please add institutional author names to references 39, 40 and others.

- Thank you and we have reviewed and added where noted in the suggested citation of these technical documents.

Please use "PLoS Med." and "PLoS ONE" as journal name abbreviations.

- Thank you and we have made these revisions.

Comments from Reviewers:

*** Reviewer #1:

My original comments were mainly to do with tidying up the presentation, and the authors have adequately responded to all of them. I have no further comments to make.

- Thank you very much for the additional review.

*** Reviewer #2:

Thank you for the opportunity to review your resubmission of the present manuscript. Appreciate the thoughtful and detailed responses raised to the initial comments provided. Overall, all major and minor comments have been addressed satisfactorily. Additionally, the authors also sufficiently address and discuss the complexities and nuances in the extant stigma literature, which have allowed for the present work to be clearly situated and therefore understood in often diverging definitions/approaches to stigma. The scope of the work is also clarified in this iteration of the manuscript. I only have a few minor comments left at this point:

Line 103-104: "Perceived stigmas refer to felt experiences of stigma or discrimination and the perception of bias4 as understood by a person living with a stigmatized identity" - I appreciate the clarifications made by the authors in defining all the forms of stigma in the revision. I would be careful with the use of the word "experiences" here as it may be confused with "Experienced Stigma". Furthermore, 'felt... discrimination' would also be somewhat confusing because discrimination already implies an action that has been done unto the stigmatized individual. Overall, removing "...experiences of..." and "or discrimination" from this sentence would suffice.

- Thank you and we have made this revision as suggested.

Line 106: "... discriminatory acts by someone." - perhaps adding something to the effect of "...on the basis of a stigmatized identity." would help clarify this.

- Thank you and we have made this revision as suggested.

Line 143: "interpretating" --> "interpreting"

- Thank you for highlighting this and we have corrected this grammatical error

*** Reviewer #3:

The authors provided detailed point-by-point responses. They substantially improved the overall clarity of their paper, and in particular, the clarity of the Methods section. They also strengthened the paper by providing an extensive overview of the limitations in the Discussion section, and outlined the opportunities for improvement in the future in a more straightforward way. The alignment of different parts of their manuscript seems much better in this version.

- Thank you very much for the additional review.

*** Reviewer #5:

Thank you for your each and every response to my comments of the first review. The second version is satisfied for further proceedings but some minor revisions are suggested as the followings:

For Table 2,

(1) There is sign of " 3 asterisks" in the foot note but the data or text referred was not found within the table.

- Thank you and please note the 3 asterisks “***” associated with the indicator: Percentage of people living with HIV who have been refused employment or a work opportunity because of their HIV status in the past 12 months***, within the Discrimination experienced in employment sub domain.

(2) The indicator of sub-Domain "Discrimination anticipated in health care settings" is needed to be numbered as "19" and the domain "Anticipated stigma" is good to be mentioned before the "Experienced stigma" domain. Therefore the order of three indicators under "Experienced stigma" domain will be 20,21 and 22.

- Thank you and we have made the changes as suggested.

In the line 346, the asterisk included in the tile of Fig 3 should be removed because the color legend has shown for the figure.

- Thank you and we have removed the asterisk in the title of Fig 2 and 3.

For Fig 3, the color legend as "Countries with sufficient available data" may be better to be complete.

- Thank you and we have revised this for both Fig 2 and Fig 3.

We appreciate the additional review and comments from the reviewers and editors. Please let us know if there are any additional updates or clarifications needed.
